# Methods to handle missing values and missing individuals

**DOI:** 10.1007/s10654-018-0461-1

**Published:** 2018-11-14

**Authors:** Carl Bonander, Ulf Strömberg

**Affiliations:** 1 Health Metrics Unit, Institute of Medicine, Sahlgrenska Academy at University of Gothenburg, PO Box 463, 405 30 Gothenburg, Sweden; 2 Department of Research and Development, Region Halland, Halmstad, Sweden

“It’s a misery” some epidemiologists may think when facing data with a lot of missing values. We are delighted that Choi and colleagues guide us on how to handle missing data in the context of propensity score analysis [1]. They addressed situations with much missing data on a covariate that acts as a confounder or an effect modifier, and simulated effect estimates by propensity matching/weighting in complete case, missing indicator, and multiple imputation analyses. In this Commentary, we provide an empirical example and extend the focus to methods for handling missing individuals (i.e. non-participants)—in addition to missing covariate data on the study participants.

## Empirical example

### Study setting and data

Choi et al. framed a cohort setting of patients providing baseline data on two continuous covariates, *X*_1_ (no missing values) and *X*_2_ (50% missing values), a binary treatment *T* and continuous outcome *Y*. We consider a somewhat different setting: a population-based cohort of individuals examined at baseline, yielding extensive data for analyzing the effect of an exposure on a binary outcome. More precisely, we consider data for estimating the association between non-alcoholic fatty liver disease (NAFLD; the exposure of interest, with an overall prevalence of 10.4%) and a positive coronary artery calcification score (CACS > 0; the outcome of interest, with an overall prevalence of 41.1%), collected at baseline examinations of a cohort of 1111 50–64 year old men and women [2]. To focus our discussion, we tone down concerns about cross-sectional data and unmeasured confounding (assuming unconfoundedness after propensity score matching/weighting).

### Missingness

We consider different types of missingness for the cohort study at issue. First, 4.8% of the individuals recruited to the cohort were ruled out according to rational exclusion criteria (related to secondary steatosis) and 3.9% were excluded because of missing data on NAFLD or CACS [2]. Furthermore, 220 individuals (19.8%) had missing covariate data on physical activity or alcohol intake. We also point out that totally 2243 individuals were invited to a baseline examination, but 1132 individuals did not participate [3]. One may argue that a large proportion (50.5%) of individuals are missing, having in mind the targeted cohort that would have been more optimal. When cohort participation is non-random, as in this population-based study [3], selection bias [4] may be enforced. We come back to this problem at the end of this Commentary.

### Re-analysis

The report by Choi et al. helped us to handle more accurately the missing data on physical activity and alcohol intake. We here focus on physical activity, which was more influential. Previously, one of us (US) handled the missing values on physical activity (n = 133) by incorporating a missing indicator variable in the exposure propensity score model [2]. As pointed out by Choi et al., bias can occur if (1) the effect is heterogeneous and (2) the missingness pattern is related to this heterogeneity. The association between NAFLD and CACS was pronounced for individuals with 0–3 metabolic risk factors present but not for individuals with more (4–7) metabolic risk factors present [2]. Individuals with high physical activity generally have fewer metabolic risk factors than those with low physical activity. Furthermore, missing values on physical activity were less common among individuals with fewer metabolic risk factors. In the subgroup of individuals with 0–1 metabolic risk factors present, 68 out of 614 individuals (11%) did not report their physical activity. The corresponding figures in the subgroups with 2–3 and 4–7 metabolic risk factors are 49/327 (15%) and 16/74 (22%), respectively, indicating that the data were not missing completely at random (MCAR). Hence, data on physical activity were either missing at random (MAR) or missing not at random (MNAR).

Sex, age, education, BMI, smoking status, waist circumference, visceral fat area and physical activity were included as covariates in the exposure propensity score model. We estimated the odds ratio (OR) for the association between NAFLD and CACS. The OR reflecting the average exposure effect for the exposed was estimated by propensity weighting, using weight 1 for each exposed individual *i* and weight *propensity score*_*j*_/(1 − *propensity score*_*j*_) for each unexposed individual *j* [5, 6]. Missing data on physical activity were handled by using the missing indicator and multiple imputation methods. The latter method included all covariates, NALFD, and CACS, as well as interaction terms between all included variables, as recommended by Choi et al.

### Results

We obtained a somewhat higher OR from the complete case analysis, as compared with the missing indicator and multiple imputation analyses (Table 1). We expected a positively biased OR from the complete case analysis, given the aforementioned effect modification by metabolic risk burden and its relation to the covariate missingness pattern. To illustrate such bias more clearly, we inflated the missingness pattern by increasing the proportions of missing values on physical activity from 15 to 68% and 22 to 72% in the subgroups of individuals with 2–3 and 4–7 metabolic risk factors present, respectively. This scenario implied a severe bias in the average effect estimate provided by the complete case analysis (Table 1). The estimator based on multiple imputation was robust to such changes. This robustness should be expected, since the missingness pattern was inflated under the MAR assumption.

**Table 1:** Odds ratio (OR) estimates reflecting the average effect of non-alcoholic fatty liver disease (NAFLD) on the odds of having a positive coronary artery calcification score among individuals with NAFLD, using different methods to handle missing values on the covarite physical activity

Method	With obtained missingness pattern		With hypothetically inflated missingness pattern	
	OR (95% CI^a^)	n^b^	OR (95% CI^a^)	n^b^
Complete case	2.33 (1.36, 4.00)	882	4.20 (1.86, 9.50)	669
Missing indicator	2.00 (1.23, 3.26)	1015	1.87 (1.15, 3.05)	1015
Multiple imputation	2.04 (1.26, 3.30)	1015	2.05 (1.26, 3.33)	1015

^a^Confidence intervals based on robust (sandwich) standard errors

^b^Number of individuals that contributed with data to the estimation

## Methods to handle missing individuals

Missing covariate data may give rise to sample selection bias. As noted by Choi et al., a “complete case estimate” reflects the average effect among the individuals selected for the analysis, i.e. those without missing values (*R *= 0); let E[*Y*_1*i *_− *Y*_0*i*_*|R *= 0] denote this effect. In the presence of sample selection bias, we generally envision a systematic discrepancy between the sample average causal effect and the average causal effect in the target population. Even in the absence of missing values on covariates, sample selection bias may be an issue of concern. A “participating case estimate” based on the participants (*P *= 0; *P* is the *non*-participant indicator) should reflect the effect E[*Y*_1*i *_− *Y*_0*i*_| *P *= 0]. Commonly, epidemiologists face both incomplete participation in a cohort study and missing covariate values for the participants. If we are interested in estimating the population average exposure effect, and there is effect heterogeneity conditional on *P*, *R*, or both, then the estimate of E[*Y*_1*i *_− *Y*_0*i*_*|P *= 0, *R *= 0] may suffer from poor validity. In relation to our example, it is easy to imagine a biased estimate of the population average exposure effect if the distributions of metabolic risk factors differ between the participants and the target population. Hence, there is an interrelation between the problem with missing individuals and the one with missing covariate values. Nevertheless, we need to handle these two problems differently. We cannot resort to multiple imputation without any data on the non-participants (or, more precisely, without any data generated by the cohort study). There are other methods to adjust for selective participation that might be useful: probability-of-participation weighting with weights derived from logistic regression or generalized boosted models based on external data on non-participants [7, 8]; post-stratification or generalized regression using known population moments [9]; entropy balancing [10]; and empirical balancing calibration weighting [11]. We welcome further investigations, in the spirit of Choi’s study, which provide guidance on methods to handle both missing individuals and missing values in the context of propensity score analysis for cohort studies.
